# The internal and external cost of motor vehicle crashes

**DOI:** 10.1038/s41598-025-89058-1

**Published:** 2025-02-14

**Authors:** Shian Dai, Liqiang Yu, Zhaoran Liu, Mengying Cui, David Levinson

**Affiliations:** 1 Intelligent Construction and Environment College, Xi’an Jiaotong University City College, Xi’an, Shaanxi Province China; 2 China Northwest Architectural Design and Research Institute Co., Ltd, Xi’an, Shaanxi Province China; 3 Key Laboratory of Transport Industry of Big Data Application Technologies for Comprehensive Transport, Beijing Jiaotong University, Beijing, China; 4 Institute of Comprehensive Transportation, National Development and Reform Commission, Beijing, China; 5 School of Transportation Engineering, Chang’an University, Xi’an, China; 6 School of Civil Engineering, The University of Sydney, Sydney, Australia

**Keywords:** Motor vehicle crashes, Internal versus external cost, Fine geographical level, Spatial variations, Civil engineering, Sustainability

## Abstract

Crash cost estimates are essential for evaluating road safety management policies and assessing the economic benefits of safety improvements. Existing studies often rely on aggregated crash data, assuming an even distribution of incidents, which overlooks significant spatial variations influenced by road characteristics and traffic conditions. This research presents a methodological framework for link-based crash cost analysis that considers both internal and external costs, enabling detailed quantification at a localized level. By employing safety performance functions and ordered probit models, we estimate on-road crash rates by crash type and injury severity, distinguishing between internal costs borne by individuals involved in crashes and external costs that impact victims, insurers, and government agencies. This framework is applied to the Minneapolis-St. Paul metropolitan area for a proof-of-concept. Our findings reveal that the costs incurred by drivers are higher than those imposed on others, and that highways are generally safer than surface streets. However, these crash costs are too low compared to the value of travel time to significantly influence route choices, even when drivers are aware of these costs. To enhance effective decision-making, related policies should consider offering incentives for safe driving practices. Future research on the practical applications of this framework is encouraged to maintain a dynamic dataset that reflects ongoing changes in road safety conditions.

## Introduction

Crash cost estimates reflect the effects of road safety management policies and allow the appraisal of the economic benefits of road safety improvement projects^1,2^. However, estimating crash costs is complex due to the involvement of various factors, including direct cost (such as property damage, medical, and legal costs), indirect cost (such as congestion, productivity loss for work and family, and tax losses), and intangible cost (such as loss of life or degradation in quality of life, and the pain and suffering for both victims and their families)^3–7^. External cost estimation is particularly challenging, as it requires consideration of multiple stakeholders, including victims, insurance companies, government agencies, and affected individuals such as families and friends^8^.

Previous studies on crash cost estimates have largely focused on the magnitude, aggregated values from large-scale crash data, and provided an overall compilation of the estimates without exploring the finer details . For instance, Blincoe et al.^9^ and Wijnen et al.^1^ estimated that national-level crash costs range from 0.4% to 4.1% of Gross Domestic Product (GDP) in European countries and the United States. Similarly, a global comparison of crash fatality costs held the same trends, but further indicates that less-developed countries have a lower percentage of GDP attributed to crash costs^10^. Levinson et al.^11^ calculated crash costs per vehicle-kilometer-traveled (vkt), reporting $0.04/vkt for rural highways and $0.02/vkt for urban highways. This supports previous observations that rural areas suffer a higher crash costs^12,13^. Parry^14^ examined external costs borne by others, finding an average of 2.2–6.6 cents per mile annually for crashes with and without fatalities during 1998–2000. However, these studies often assume an even distribution of crashes and fail to capture spatial variations across road networks.

In reality, crash costs are influenced by road characteristics and traffic conditions, leading to significant fluctuations at a fine geographical scale^15–17^. The principles of “Sustainably-Safe Traffic” emphasize the importance of encouraging drivers to select safer routes to reduce crash casualties^18,19^, which requires more localized estimates of crash cost differences. Unfortunately, this aspect has not been adequately solved in previous research.

To address these gaps, this study proposes a methodological framework for link-based crash cost analysis that considers both internal and external cost factors and explains how to quantify the average and marginal values at a microscopic level. It allows for an examination of spatial variations in crash costs, identifying which road segments are safer for travel.

As a proof-of-concept, this framework is applied to the Minneapolis-St. Paul (Twin Cities) metropolitan area to assess crash costs across the region’s road network. The data, methodology, results, and conclusion of this study are discussed in sections Methodology–Conclusions in turn.

## Methodology

**Fig. 1:**
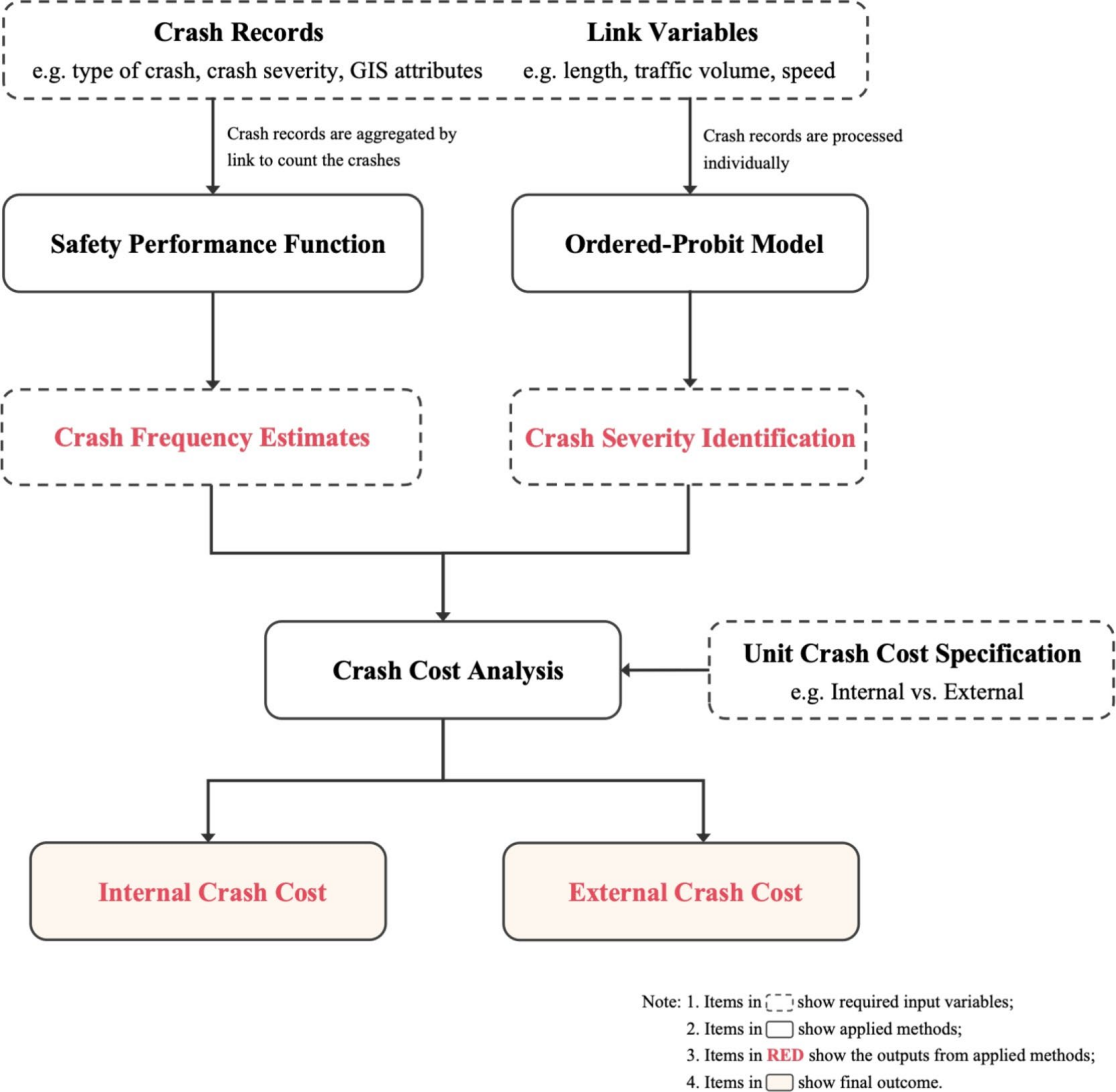
Methodological framework: internal and external crash cost estimates.

The National Safety Council (NSC) introduced the “KABCO” injury scale to classify crash severity levels: “K” for fatal crashes, “A” for incapacitating injuries, “B” for non-incapacitating injuries, “C” for pain complaints, and “O” for property-damage only crashes. This scale has been widely adopted for reporting crash records and establishing crash cost estimates^20^. Previous studies typically estimated overall crash frequency without distinguishing crash types initially, and then analyzed crash severity levels separately^21–24^. These estimates can be further used for crash cost analysis by applying unit crash cost specifications to monetize the results. These steps build the train of thoughts in this study, as outlined in Fig. 1.

This framework can be applied to any region where the necessary data, including crash records and link-related variables, are accessible. Detailed methodologies are presented in the following subsections.

### Crash frequency

Safety Performance Functions (SPFs), as defined in Highway Safety Manual (HSM)^25^, are statistical base models that can be used to estimate the average crash frequency based on specific roadway features under existing conditions or to predict crash frequency under projected future conditions^26^. SPFs are applicable at both the micro (e.g., individual road segments or intersections) and the macro level (e.g., transportation analysis zones or counties)^27^.

In this study, micro-level estimation is performed, considering individual link segments as observations. The resulting models are applied to all links across the metropolitan area. Notably, conventional variables used in SPFs include Annual Average Daily Traffic (AADT) and segment length. However, additional variables, such as those representing driving speed or speed variance, are also tested to improve model performance.

We use the Negative Binomial Distribution for the SPFs, which is an extension of the Poisson Distribution that effectively models count data with overdispersion (where variance exceeds the mean)^26^. This overdispersion often arises from the nature of crash occurrences, as many roads report a frequency of zero crashes, while only a few locations experience multiple incidents. Traditional models may underestimate the variability in such cases, leading to inaccurate representations of crash patterns.

The Negative Binomial model for SPFs is expressed as follows,

1 $$\begin{aligned} ln(y)=\beta _{0}+\sum _{k=1}^{K}\beta _{k}x_{k} \end{aligned}$$

where *y* represents the dependent variable measuring the number of crashes in SPFs, $x_{k}$ represents independent variables, *K* represents the number of independent variables, $\beta _{k}$ are coefficients.

### Crash severity

The ordered probit model is widely used for crash severity analysis and is suitable for cases involving categorical dependent variables^28–30^. Specifically, it recognizes the ordinal nature of the data and operates under the assumption of a continuous underlying variable with normally distributed errors, enabling more efficient parameter estimation while reducing the number of required parameters. Moreover, this model effectively addresses the limitations found in traditional approaches, such as the independence of irrelevant alternatives and the lack of a closed-form likelihood^30,31^.

Its general specification is expressed as:

2 $$\begin{aligned} y^{*}_{j}=X_{j}\beta +\varepsilon _{j} \end{aligned}$$

where $y^{*}_{j}$ is a latent variable describing the crash severity of the $j^{th}$ crash, $X_{j}$ is a vector of independent variables, $\beta$ is a vector of coefficients, $\varepsilon _{j}$ is the random error term.

The observed variable $y_{j}$ is resolved by the following model,

3 $$\begin{aligned} y_{j}= \left\{ \begin{array}{l l} 1 & \quad {if \ \ -\infty \ \le y^{*}_{j} \le \ \mu _{1}} \\ 2 & \quad {if \ \ \ \mu _{1} \le y^{*}_{j} \le \ \mu _{2}}\\ 3 & \quad {if \ \ \ \mu _{2} \le y^{*}_{j} \le \ \mu _{3}}\\ 4 & \quad {if \ \ \ \mu _{3} \le y^{*}_{j} \le \ \mu _{4}}\\ 5 & \quad {if \ \ \ \mu _{4} \le y^{*}_{j} \le \ \infty }\\ \end{array}\right. \end{aligned}$$

where $y_{j}=(1,2,3,4,5)$ stand for difference severity levels, recognizing property damage only, complaint of pain, non-incapacitating injury, incapacitating injury, and fatal crashes, respectively; $\mu _{1}$, $\mu _{2}$, $\mu _{3}$ and $\mu _{4}$ stand for the to-be-estimated threshold values.

### Unit crash cost specifications

From a traveler’s perspective, crash costs include both internal and external components. Internal costs refer to the expenses borne by each traveler involved in a crash, while external costs account for the impact on others, such as victims, insurance companies, and government agencies^8^.

Blincoe et al.^9^ evaluated the economic costs of motor vehicle crashes by examining various cost factors based on the severity of incidents. These cost factors include direct expenses, such as medical expenses, property damage, legal fees, and vehicle repairs, as well as indirect costs like lost productivity, insurance premiums, and administrative expenses. However, a key question remains: to what extent are these cost factors external to individual travelers?

Vickrey^32^ defined crash externality as the increase in crash costs experienced by existing drivers due to additional vehicles on the roads. This definition simplifies the analysis by bypassing the complexities of joint effects, such as driving behavior, insurance policies, and traffic laws. Empirical studies have shown that marginal changes in crash costs can sometimes be negative, as increased congestion raises crash rates but lowers severity due to slower speeds^33,34^.

Parry^14^ quantified the external portion of various cost factors for single- and multi-vehicle crashes, providing a foundation for subsequent crash cost studies^8,35,36^. In our study, we define unit crash cost specifications using estimates from Blincoe et al.^9^ as a reference, see Table 1. This table outlines the cost factors considered in our crash cost estimates and the external proportion of each cost factor based on Parry^14^’s study.

In summary, in our estimates,

- The internal costs of crashes fully account for lost productivity, both in the market and household settings, and partially include medical expenses, property damage, emergency service costs, insurance administration, and legal fees. The proportions of these costs are determined by individual health and vehicle insurance policies; however, we use an aggregated average here. The loss of quality of life is considered as an internal cost if the incident involves a single vehicle; in the case of multi-vehicle crashes, it is only partially accounted for as an internal cost.
- The external costs of crashes include the remaining medical expenses, property damage, emergency service costs, insurance administration, legal fees, and the loss of quality of life in multi-vehicle incidents. This category also fully accounts for workplace disruptions due to employee loss or absence, as well as congestion costs resulting from vehicle crashes.

Based on the unit crash cost specifications, the average internal and external crash costs can be estimated as follows:

4 $$\begin{aligned} C_{\bar{s},i_{f,Q}}=\sum _{z} \frac{N_{s,i_{f,Q}}*R_{s,i_{f,Q}}*u_{s_{z}}}{N_{Y}*N_{D}*Q} \end{aligned}$$

where $C_{\bar{s},i_{f,Q}}$: Average crash cost on link $i_{f}$, where *f* specifies the functional road classifications and *Q* refers to the average annual daily traffic (AADT), defining the traffic condition; $N_{s,i_{f,Q}}$: Expected crash frequency on link $i_{f}$; $R_{s,i_{f,Q},z}$: Probability of crashes specific to severity level *z* happened on link $i_{f,Q}$; $u_{s_{z}}$: Unit crash cost, internal or external, specific to severity level *z*; $N_{Y}$ and $N_{D}$ describe the duration of the analysis period, representing the number of years in counting and number of days in a year, respectively.

Marginal crash costs are calculated by introducing one additional vehicle on each link to evaluate the combined effects of changes in crash frequency ($N_{s,i_{f,Q}}$) and severity ($R_{i_{f,Q}},z$), expressed as:

5 $$\begin{aligned} C_{\hat{s},i_{f,Q}}=(C_{\bar{s},i_{f,Q+1}}-C_{\bar{s},i_{f,Q}})\times Q \end{aligned}$$

**Table 1:** Unit crash cost specification and their external portion. (*Source*: Blincoe et al.^9^ and Parry^14^).

Crash cost factor	Definition	External proportion	Fatal	Incapacitating injury	Non-incapacitating injury	Complaint of pain	Property-damage only
Medical	The cost of medical treatment related to traffic crash injuries	85%	$11,317	$21,189	$4,981	$4,393	$2,571
Property damage	The value of vehicles or other items damaged in traffic crashes	50%	$10,712	$3,518	$2,465	$2,407	$1,624
Market	The discounted value of the lost wages and benefits over the victim’s remaining life span	0%	$933,262	$24,403	$6,465	$5,096	$2,184
EMS	Emergency service cost	85%	$902	$122	$56	$45	$20
Household productivity	The value of lost productive household activity	0%	$289,910	$7,182	$1,966	$1,562	$710
Insurance administration	The administrative expenses of processing insurance claims due to motor vehicle crashes	85%	$28,322	$11,751	$3,670	$3,648	$2,240
Workplace cost	The cost of workplace disruption due to the loss or absence of an employee	100%	$11,783	$3,941	$1,459	$208	$7
Legal cost	The legal fees and court costs associated with civil litigation resulting from traffic crashes	85%	$106,488	$8,557	$1,684	$1,125	$56
Congestion cost	The costs due to congestion results from motor vehicle crashes	100%	$5,720	$1,385	$995	$1,009	$1,026
Quality of life	Lost quality of life	0% for single-vehicle crashes; 1/(n − 1) of all injuries in a crash.	$7,747,082	$919,158	$252,268	$108,274	$31,859

## Data

**Fig. 2:**
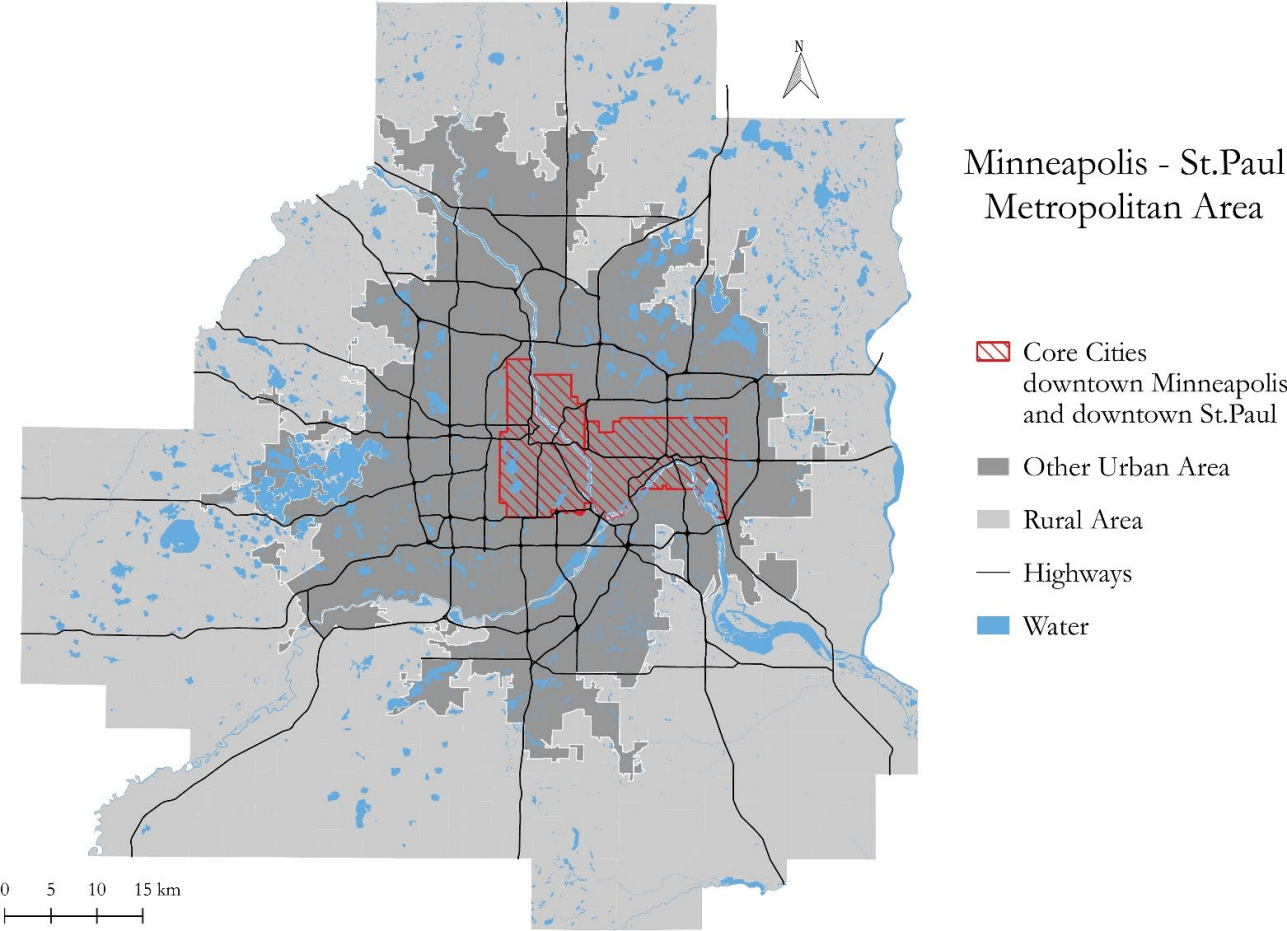
Map of the Minneapolis - St. Paul (Twin Cities) Region.

This study incorporates multiple datasets to implement the proposed framework in a real-world scenario, as outlined in the process shown in Fig. 1. The geographical scope of the study area, the seven-county Minneapolis-St. Paul Metropolitan Area, is shown in Fig. 2.

### Crash records

Crash records from 2003 to 2014 were obtained from the Minnesota Department of Transportation (MnDOT). Note that these records track police-reported crashes, which are more reliable for documenting severe crashes but likely under-report minor ones. Throughout the remainder of the text, crash data refers only to reported crashes.

The records for each year include GIS attributes, e.g., route numbers, reference points, and coordinates, along with crash-related details like type, severity, weather conditions, and lighting. These records are provided as GIS shapefiles, enabling precise mapping onto the road network. The data can be aggregated by link segment to calculate crash counts for use as dependent variables in safety performance functions or analyzed individually to examine crash severity for ordered probit models.

Table 2 summarizes the number of crashes, categorized by crash type, severity, and Functional Road Classification, over the 12-year period.

**Table 2:** Total number of crashes (12 years) by crash type, severity, and functional road classification. (*Source* Minnesota Department of Transportation).

Severity	Single-vehicle crashes				
	Primary arterial	Minor arterial	Collector	Local	Total
Fatal	154	191	67	29	441
Incapacitating injury	433	683	285	155	1556
Non-incapacitating injury	3477	2816	1107	602	8002
Complaint of pain	7090	4775	1690	960	14,515
Property damage only	27,786	21,275	7124	4207	60,392
Total	38,940	29,740	10,273	5953	84,906

Severity	Multi-vehicle crashes				
	Primary arterial	Minor arterial	Collector	Local	Total
Fatal	286	513	123	39	961
Incapacitating injury	841	2724	818	424	4807
Non-incapacitating injury	7182	15,685	4856	2348	30,071
Complaint of pain	26,385	43,057	12,950	6,266	88,658
Property damage only	90,283	132,502	43,932	23,673	290,390
Total	124,977	194,481	62,679	32,750	414,887

### Link variables

The TomTom road network, sourced from the Metropolitan Council, is drawn as a polyline shapefile that provides spatial details of roadways within the Twin Cities network^37^. Variables such as link length and road type are derived directly from this dataset.

TomTom speed data offers speed estimates aggregated from millions of GPS records and linked to the TomTom road network. These estimates are stratified by time periods, dividing a day into seven parts to account for peak and non-peak hours, as well as by speed percentiles, ranging from the fastest 5% to the slowest 5% of recorded speeds. For this study, the 50th percentile (median) speed during morning peak hours (7 a.m. to 9 a.m.) is selected to represent travel speed, while the difference between the 10th and 90th percentiles is used as an indicator of speed variance. These measures serve as independent variables in crash frequency estimation models. Notably, the speed variance reflects an aggregated yearly index across all vehicles using the link, rather than intra-day or intra-vehicle variations.

The MnDOT Traffic Volume Program^38^ provides Annual Average Daily Traffic (AADT) estimates for Minnesota, based on data collected from approximately 33,000 count locations on trunk highways, county state aid highways (CSAH), county roads (CR), and municipal state aid streets (MSAS). Traffic counts, typically recorded over short durations (e.g., 48 hours), are adjusted using seasonal and axle correction factors (for trunk highways). As a standard independent variable in safety performance functions, AADT data for the Twin Cities metro region was extracted and integrated with the TomTom road network.

The Federal Urban/Rural GIS Shapefile, obtained from MnDOT’s Transportation Data and Analysis division^39^, delineates roadways in Minnesota by Federal Adjusted Urban Area boundaries into Urban, Small Urban, and Rural classifications. This dataset is also linked to the TomTom road network for further analysis.

## Results

Separate models are developed for estimating crash frequency and severity, specific to crash type and functional road classifications, for two main reasons: first, these estimates are used in crash cost analysis, where internal and external cost assignments differ by crash type and by single- versus multi-vehicle crashes; second, using specialized models for different road types is statistically validated, as road types have distinct attributes influencing crash characteristics^23,40^.

### Safety performance function

The selected independent variables for the SPFs are described in Table 3. Note that $V_{\text {Var}}$ is not the typical measure of speed variance, which reflects the dispersion of space-mean speeds among drivers within or across lanes at the same time^41,42^. Instead, it is more likely to represents the dispersion of time-mean speeds, calculated as the difference between the fastest 5% and the slowest 5% speeds over a specific time period, such as morning peak hours across a year.

The regression results of the safety performance functions are shown in Table 4. Note that speed (*V*) is dropped from all models to avoid multicollinearity problem, as it is highly correlated with AADT (*Q*). AADT (*Q*) and segment length (*L*) are transformed into natural log format. Other functional forms are also tested, but cannot improve the fits.

**Table 3:** Definitions and descriptive statistics of independent variables selected for the safety performance functions.

Variables	Description	Primary arterial (n=4,663)		Minor arterial (n=16,878)		Collector (n=15,738)		Local (n=10,742)	
		Mean	S.D.	Mean	S.D.	Mean	S.D.	Mean	S.D.
N_S_	Dependent Variables, number of single-vehicle crashes on a link	8.37	22.58	1.78	4.15	0.66	1.84	0.57	1.60
N_M_	Dependent Variables, number of multi-vehicle crashes on a link	26.82	70.24	11.53	26.46	3.99	12.33	3.05	9.41
Q	AADT (vehicles)	39,591	42,062	9,930	15,219	6,050	14,824	7,735	18,215
L	Length of link segment (km)	0.62	0.71	0.59	0.67	0.41	0.47	0.47	0.54
V	Median speed, specifically the 50th percentile TomTom speed	73.83	23.76	56.52	17.45	52.54	12.90	50.49	12.43
V_Var_	Speed variance, specifically the differences between the 10th and 90th percentile TomTom speed	30.42	15.41	25.57	10.63	23.01	9.50	22.30	10.47
U	Dummy variable, urban road=1, others=0	0.84	0.36	0.80	0.40	0.81	0.39	0.82	0.38

**Table 4:** Safety performance function results for single-vehicle and multi-vehicle crashes by roadway class.

		Primary arterial			Minor arterial			Collector			Local roads		
		Est.	S.E.		Est.	S.E.		Est.	S.E.		Est.	S.E.	
Single-Vehicle Crashes	Const.	0.715	0.199	***	− 1.226	0.095	***	− 2.273	0.111	***	− 1.884	0.145	***
	ln(Q)	0.052	0.019	**	0.221	0.011	***	0.306	0.014	***	0.182	0.016	***
	ln(L)	0.640	0.031	***	0.813	0.015	***	0.820	0.018	***	0.683	0.024	***
	V_Var_	0.019	0.002	***	0.001	0.001		0.000	0.002		0.002	0.002	
	U	0.811	0.088	***	0.466	0.036	***	0.314	0.046	***	0.491	0.059	***
	AIC	23,461			53,505			30,417			19,693		
	Pseudo $R^{2}$	0.0217			0.0621			0.0743			0.0484		
Multi-Vehicle Crashes	Const.	0.726	0.196	***	− 2.831	0.100	***	− 5.728	0.122	***	− 2.897	0.142	***
	ln(Q)	0.127	0.019	***	0.463	0.012	***	0.784	0.015	***	0.421	0.016	***
	ln(L)	0.445	0.030	***	0.347	0.013	***	0.443	0.017	***	0.470	0.021	***
	V_Var_	0.019	0.002	***	0.006	0.001	***	0.002	0.002		0.006	0.002	***
	U	1.126	0.085	***	1.487	0.038	***	1.220	0.049	***	0.981	0.056	***
	AIC	32,128			99,052			59,135			39,300		
	Pseudo $R^{2}$	0.0147			0.0317			0.0537			0.0304		

***p-value<0.001, **p-value<0.01, *p-value<0.05, . *p*-value <0.1

.

The regression results for the SPFs are presented in Table 4. Note that speed (*V*) is excluded from all models to avoid multicollinearity problem, as it is highly correlated with AADT (*Q*). Both AADT (*Q*) and segment length (*L*) are transformed into their natural logarithmic forms for better model performance. Alternative functional forms were tested but did not improve the model fits.

The conventional variables (*Q* and *L*) have significant positive effects on crash counts for both single- and multi-vehicle crashes across all road classifications. As expected, links with higher AADT or longer lengths experience more crashes, regardless of the crash type. Speed variance , which indicates on-road shockwaves, is positively correlated with crash counts, highlighting that more severe stop-and-go driving conditions are associated with higher collision rates, particularly for multi-vehicle crashes.

Additionally, urban roadways tend to have higher crash counts than rural ones. This pattern may be attributed to network structure features, such as higher road density in urban areas.

### Ordered probit model

The same link property attributes are used as independent variables in the ordered probit models to estimate the probability of each injury severity category for a given crash. Additional dummy variables are included to capture road surface conditions, including $W_{\text {Wet}}$, $W_{\text {Snow}}$, $W_{\text {Iced}}$, and $W_{\text {Others}}$, with dry road surface serving as the baseline. To account for light conditions, two dummy variables are added to identify the road light-on ($D_{\text {light-on}}$) and light-off ($D_{\text {light-off}}$) scenarios, using daylight as the reference group. The descriptive statistics for these variables are summarized in Table 5.

As in the safety performance functions, the natural logarithmic transformations of *Q* and *L* are used, as they yield lower AIC values and higher pseudo $R^{2}$, compared to the untransformed variables. The regression results for the ordered probit models are shown in Table  6.

The analysis reveals that AADT is negatively correlated with crash severity, indicating that higher traffic volumes are associated with less severe crashes, likely because drivers tend to exercise greater caution on busier roadways. Segment length shows a positive relationship with crash severity, as longer segments are associated with more severe crashes. Speed variance has some impact, particularly in cases such as multi-vehicle crashes on primary and minor arterials; however, the coefficients are too small to significantly affect the probability of each injury severity category. This finding is surprising, as greater speed variance was expected to have a stronger positive effect, particularly for multi-vehicle crashes. Future studies should consider this question. Additionally, urban roadways are associated with less severe crashes, likely due to lower speeds and shorter travel distances in urban environments.

The road surface condition variables show that crashes on wet, snowy, and icy roads are associated with lower injury severity compared to crashes on dry roads. These results align with previous findings that adverse winter conditions, such as snow and ice, tend to reduce injury severity due to lower average speeds and more cautious driving. However, property damage-only crashes are more frequent during winter, as noted in other studies^43–45^.

### Link-based crash frequency and severity estimates

Link-based crash frequency and severity, along with their marginal changes, are estimated under current traffic conditions. The mean values across different link types are summarized in Table 7. As expected, highways experience a higher number of crashes compared to surface roadways for both single- and multi-vehicle crashes; however, the proportion of injury crashes is comparatively lower, with property damage-only (PDO) crashes accounting for over 70% of the total. Urban roads generally have a higher crash frequency than rural roads, while roads in core cities, Minneapolis and St. Paul, exhibit lower crash frequencies compared to suburban areas (urbanized areas outside the core cities). The severity distribution across area types indicates that fatal crashes, for both single- and multi-vehicle types, represent a higher percentage in rural areas. Additionally, the introduction of more vehicles on roads increases crash frequency but reduces injury severity, consistent with the regression results.

**Table 5:** Descriptive statistics of independent variables selected for the ordered probit models.

	Single-vehicle crashes								Multi-vehicle crashes							
	Primary arterial (n=38,703)		Minor arterial (n=29,303)		Collector (n=10,145)		Local roads (n=5,884)		Primary arterial (n=124,986)		Minor arterial (n=194,537)		Collector (n=62,712)		Local roads (n=32,770)	
	Mean	S.D.	Mean	S.D.	Mean	S.D.	Mean	S.D.	Mean	S.D.	Mean	S.D.	Mean	S.D.	Mean	S.D.
Q	51,944	47,362	11,518	17,148	8,336	21,168	10,611	22,481	57,983	53,665	14,815	18,785	10,573	23,029	10,940	22,259
L	1.130	1.334	1.149	1.126	0.762	0.682	0.812	1.187	0.822	0.866	0.587	0.552	0.464	0.398	0.619	0.898
V	87.427	20.471	63.201	20.071	53.777	16.522	50.997	14.664	80.850	21.722	51.596	17.032	46.494	14.254	47.319	14.589
V_Var_	33.748	17.666	25.197	10.981	23.022	10.993	23.028	12.018	35.200	17.885	27.014	11.444	23.955	12.748	23.378	12.534
U	0.901	0.299	0.801	0.399	0.827	0.378	0.865	0.342	0.945	0.229	0.951	0.216	0.962	0.192	0.933	0.250
W_Wet_	0.171	0.376	0.127	0.332	0.122	0.327	0.120	0.325	0.155	0.362	0.158	0.364	0.147	0.354	0.136	0.343
W_Snow_	0.145	0.352	0.104	0.305	0.105	0.307	0.105	0.307	0.057	0.231	0.057	0.232	0.067	0.250	0.076	0.265
W_Iced_	0.160	0.367	0.134	0.341	0.147	0.354	0.148	0.355	0.061	0.239	0.063	0.243	0.081	0.272	0.098	0.297
W_Others_	0.049	0.215	0.044	0.206	0.045	0.207	0.050	0.217	0.019	0.136	0.025	0.157	0.031	0.174	0.031	0.173
D_light-on_	0.320	0.466	0.287	0.452	0.309	0.462	0.356	0.479	0.180	0.384	0.193	0.395	0.208	0.406	0.199	0.399
D_light-off_	0.103	0.304	0.186	0.389	0.158	0.365	0.110	0.313	0.026	0.160	0.027	0.161	0.035	0.184	0.039	0.193

W, dummy variables specifying road surface conditions, where dry surface is the reference group;

D, dummy variables identifying light conditions, where daylight is the reference group.

**Table 6:** Regression results of ordered probit models to estimate crash severity for single-vehicle and multi-vehicle crashes by roadway class.

			Primary arterial			Minor arterial			Collector			Local roads		
			Est.	S.E.		Est.	S.E.		Est.	S.E.		Est.	S.E.	
Single-vehicle crashes	Coef.	log(Q)	0.007	0.004	.	− 0.034	0.007	***	− 0.049	0.011	***	− 0.029	0.013	*
		log(L)	− 0.002	0.007		0.005	0.009		0.004	0.013		0.049	0.018	*
		V_Var_	− 0.000	0.000		0.001	0.001	*	− 0.002	0.001		− 0.001	0.004	
		U	− 0.036	0.023		− 0.121	0.021	***	− 0.062	0.036	.	− 0.054	0.048	
		W_Wet_	− 0.270	0.018	***	− 0.146	0.023	***	− 0.204	0.039	***	− 0.152	0.051	***
		W_Snow_	− 0.503	0.021	***	− 0.388	0.027	***	− 0.383	0.044	***	− 0.345	0.058	***
		W_Iced_	− 0.339	0.019	***	− 0.375	0.024	***	− 0.482	0.039	***	− 0.441	0.051	***
		W_Others_	− 0.330	0.032	***	− 0.083	0.036	*	− 0.100	0.059	.	− 0.122	0.075	
		D_light-on_	− 0.059	0.014	***	− 0.088	0.018	***	− 0.102	0.029	***	− 0.066	0.036	.
		D_light-off_	− 0.167	0.023	***	− 0.253	0.021	***	− 0.112	0.037	***	− 0.009	0.054	
	Const.	1|2	0.377	0.046	***	0.013	0.059		− 0.210	0.091	*	0.018	0.112	
		2|3	1.081	0.047	***	0.597	0.059	***	0.348	0.091	***	0.572	0.112	***
		3|4	1.984	0.049	***	1.282	0.060	***	1.021	0.091	***	1.210	0.114	***
		4|5	2.411	0.052	***	1.705	0.061	***	1.420	0.094	***	1.565	0.116	***
	AIC		64,572			52,530			19,534			11,185		
	R2		0.014			0.012			0.014			0.011		
Multi-vehicle crashes	Coef.	log(Q)	− 0.035	0.002	***	− 0.044	0.003	***	− 0.010	0.006	.	− 0.012	0.006	.
		log(L)	0.043	0.004	***	0.053	0.003	***	0.044	0.006	***	0.028	0.008	***
		V_Var_	− 0.001	0.000	***	0.001	0.000	***	0.000	0.000		0.000	0.001	
		U	− 0.088	0.016	***	− 0.129	0.013	***	− 0.155	0.026	***	0.091	0.029	***
		W_Wet_	− 0.041	0.010	***	− 0.069	0.008	***	− 0.099	0.014	***	− 0.105	0.021	***
		W_Snow_	− 0.245	0.017	***	− 0.370	0.013	***	− 0.452	0.023	***	− 0.439	0.030	***
		W_Iced_	− 0.145	0.016	***	− 0.424	0.013	***	− 0.442	0.021	***	− 0.469	0.027	***
		W_Others_	− 0.119	0.027	***	− 0.270	0.019	***	− 0.306	0.031	***	− 0.358	0.046	***
		D_light-on_	0.071	0.009	***	0.066	0.007	***	− 0.029	0.013	*	− 0.051	0.018	***
		D_light-off_	0.199	0.022	***	− 0.003	0.018		− 0.288	0.031	***	− 0.315	0.042	***
	Const.	1|2	0.074	0.028	*	− 0.129	0.028	***	0.130	0.051	*	0.440	0.062	***
		2|3	0.991	0.029	***	0.704	0.028	***	0.938	0.052	***	1.225	0.062	***
		3|4	1.858	0.030	***	1.539	0.029	***	1.781	0.052	***	2.049	0.064	***
		4|5	2.330	0.034	***	2.181	0.031	***	2.438	0.057	***	2.786	0.074	***
	AIC		193,076			337,992			104,853			52,308		
	R2		0.004			0.008			0.011			0.012		

***p-value<0.001, **p-value<0.01, *p-value<0.05, . *p*-value <0.1.

Order Definition: Fatal=5; Incapacitating Injury=4; Non-Incapacitating Injury=3; Complaint of Pain=2; Property Damage Only=1.

**Table 7:** Estimates of average crash frequency and severity and their marginal changes.

			By area type			By road type		All roads
			Core cities	Other urban area	Rural area	Highways	Surface roadways	
Single-vehicle crashes	Frequency		1.345	2.019	1.632	9.859	1.230	1.781
			(0.395)	(0.937)	(2.666)	(1.942)	(1.183)	(1.232)
	Severity	Fatal	2.168%	2.224%	2.797%	0.950%	2.444%	2.349%
			(− 0.612%)	(− 1.456%)	(-4.386%)	(0.066%)	(− 2.111%)	(− 1.972%)
		Incapacitating injury	3.059%	3.057%	3.700%	1.764%	3.309%	3.210%
			(− 0.609%)	(− 1.326%)	(− 3.896%)	(0.118%)	(− 1.913%)	(− 1.783%)
		Non-incapacitating injury	12.065%	12.211%	13.616%	12.148%	12.539%	12.514%
			(− 1.519%)	(− 3.069%)	(− 8.582%)	(0.637%)	(-4.367%)	(-4.047%)
		Complaint of pain	18.410%	18.663%	19.488%	21.573%	18.616%	18.805%
			(− 1.095%)	(− 1.956%)	(-4.846%)	(0.587%)	(− 2.666%)	(− 2.458%)
		Property damage only	64.297%	63.845%	60.399%	63.565%	63.092%	63.122%
			(3.835%)	(7.807%)	(21.710%)	(− 1.408%)	(11.057%)	(10.261%)
Multi-vehicle crashes	Frequency		9.654	10.667	4.782	31.911	7.486	9.045
			(6.186)	(8.679)	(9.619)	(10.321)	(8.230)	(8.364)
	Severity	Fatal	0.273%	0.285%	0.433%	0.250%	0.322%	0.318%
			(− 0.055%)	(− 0.149%)	(− 0.499%)	(− 0.269%)	(− 0.208%)	(− 0.212%)
		Incapacitating injury	1.408%	1.457%	1.955%	0.765%	1.620%	1.565%
			(− 0.206%)	(− 0.494%)	(− 1.539%)	(− 0.556%)	(− 0.689%)	(− 0.681%)
		Non-incapacitating Injury	8.172%	8.334%	10.138%	6.189%	8.902%	8.729%
			(− 0.788%)	(− 1.811%)	(-4.895%)	(− 2.768%)	(− 2.294%)	(− 2.325%)
		Complaint of pain	21.987%	22.237%	24.334%	21.725%	22.748%	22.683%
			(− 1.046%)	(− 2.215%)	(-4.886%)	(− 3.964%)	(− 2.506%)	(− 2.599%)
		Property damage only	68.161%	67.687%	63.139%	71.070%	66.408%	66.706%
			(2.096%)	(4.669%)	(11.819%)	(7.557%)	(5.697%)	(5.816%)

*Values in the bracket ($\times 10^{-4}$) refers to the marginal changes of crash frequency and severity estimates.

### Crash cost estimates

The internal and external crash costs are estimated for each link in the Twin Cities road network, considering both average and marginal costs. Weighted average costs are calculated to summarize the estimates using the following formula:

6 $$\begin{aligned} \bar{C}_{w,s}=\frac{\sum _{i}C_{s,i}*Q_{i}*L_{i}}{\sum _{i}Q_{i}*L_{i}} \end{aligned}$$

where $$\bar{C}_{w,s}$$: Weighted average crash cost; $$C_{s,i}$$: Crash cost on link *i* ($/veh); $$Q_{i}$$: AADT on link *i*; $$L_{i}$$: Segment length of link *i* (km).

The results, presented in Table 8, show that the weighted average internal crash cost across all links in the Twin Cities is approximately $0.13/veh-km, with 94.5% of links having a value below $1.00/veh-km. While costs are similar by area type, roads in core cities generally exhibit higher internal crash costs than suburban roads, and rural roads are slightly more hazardous. Highways, however, are significantly safer than other surface roadways, with a weighted average cost of about 1/2–1/3 of that for other roads.

**Table 8:** Link-based Internal and External Crash Cost Estimates ($/vkt): This table shows that the internal crash costs incurred by travelers themselves are higher than the external costs imposed on others, and that highway crash costs are lower compared to those for other road types.

		By area type			By road type		All roads
		Core cities	Suburban	Rural	Highways	Surface roadways	
Average	Internal	0.146	0.119	0.154	0.062	0.154	0.130
	External	0.095	0.072	0.082	0.030	0.097	0.079
Marginal	Internal	0.058	0.040	0.050	0.005	0.061	0.046
	External	0.045	0.030	0.034	0.003	0.046	0.034

The external average crash cost imposed on others is much lower than the internal costs borne by travelers. The weighted average external cost is approximately $0.08/veh-km, with 98% of links having an external average crash cost below $1.00/veh-km. The trends for external costs by area type and road type are the same as those for internal crash costs.

Marginal internal and external crash costs are lower than their average counterparts, but the patterns remain consistent: internal crash costs borne by travelers exceed external costs imposed on others, and highways and suburban roads (outside core cities) are safer than other roadways.

Our estimates reveal no significant conflicts with previous studies in terms of magnitude^1^. For instance, Levinson and Gillen^46^ reported an average crash cost of $0.048 per veh-km for intercity highways. Although this estimate does not specify the proportions of internal and external costs, we believe it primarily reflects the internal costs of crashes, as the factors considered mainly pertain to the functional years lost due to personal injury severity. Additionally, several studies have estimated external crash costs, e.g., Lemp and Kockelman^36^, who reported $0.077 per veh-km, and Parry and Small^47^, who reported $0.026 per veh-km in the United States. However, to the best of our knowledge, no previous studies provide fine-grained crash cost estimates that differentiate between internal and external costs by urban area and road type. Therefore, no further comparisons can be made at this stage.

**Fig. 3:**
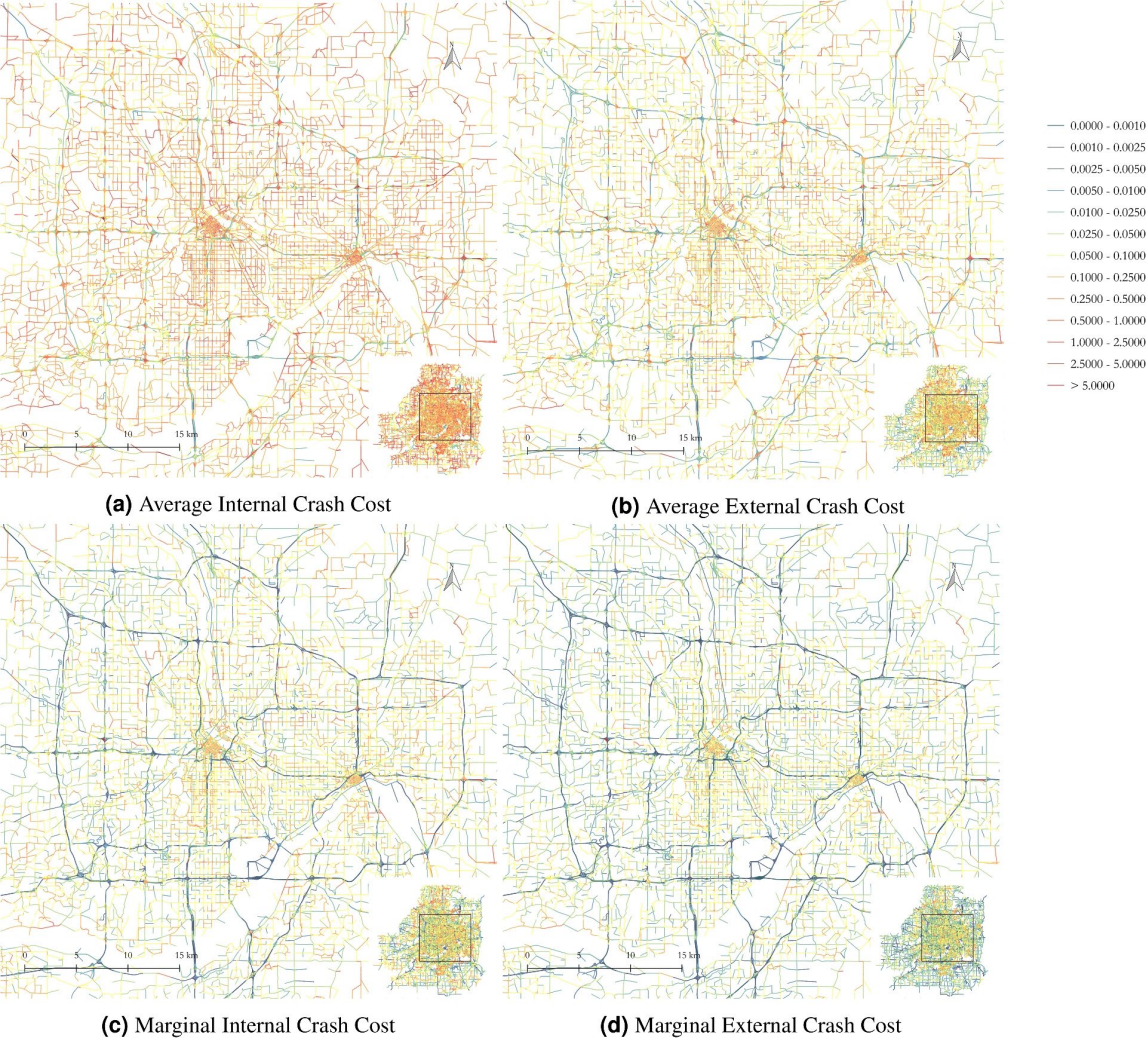
Link-based crash cost estimates for both internal and external versions ($/veh-km): highways are notable in these maps for being safer than surface roadways, as indicated by the blue shape.

Figure 3 illustrates the spatial distribution of crash cost estimates across the network. It confirms that internal crash costs exceed external costs for both average and marginal calculations (Fig. 3a vs. 3b and Fig. 3c vs.  3d). Additionally, highways are notable for being safer than surface roadways, as indicated by the blue shape in the network maps.

## Conclusion

This study develops a framework for link-based crash cost analysis, which quantifies the internal and external costs of vehicle crashes at a detailed, microscopic level and implements these costs across a metropolitan road network. To be more specific, internal costs refer to those borne by travelers involved in crashes, while external costs are those imposed on others, such as victims, insurance companies, or government agencies.

The framework is applied to the Twin Cities region as a case study. The results indicate that the weighted average internal crash cost is approximately $0.130/veh-km, while the average external crash cost is about $0.079/veh-km. This demonstrates that travelers bear significantly higher crash costs themselves compared to what they impose on others. Importantly, highways exhibit lower average internal and external crash costs compared to surface roadways, reinforcing the conclusion that they are safer due to their effectiveness in reducing crash-related costs, in line with 2022 statistics from the Insurance Institute for Highway Safety^48^. This finding suggests that superior design standards of highways, such as separation of traffic, access control, and surface configurations, enhance both travel efficiency and safety. Therefore, allocating resources to upgrade existing roadway infrastructure to higher standards could effectively reduce the incidence of crashes and enhance overall safety in urban areas. However, specific investments should be guided by detailed benefit-cost evaluations, consideration of government budgets, and alignment with strategic plans, particularly if other safety management projects need to take priority for greater efficiency.

Given a value of time at $18.30/hr ($0.305/min)^49^, which is about twice the average internal crash cost and three times the external cost (based on the network’s mean 50th percentile speed of 62.58 km/hr), crash costs appear to play a smaller role than travel time in route decisions, even if travelers were aware of these costs^50^. However, insurance companies could play a crucial role by offering incentives for safe driving practices. For instance, reduced premiums or rewards for drivers who maintain a clean driving record could encourage more cautious behavior behind the wheel. But this approach raises important technical questions about how to keep link-based datasets dynamically updated and effectively communicate this information to drivers. If these challenges can be addressed, a positive feedback loop could be established: as dangerous routes are improved and become safer, the updated estimates would reflect these changes, potentially altering drivers’ route preferences even further. Policymakers may also benefit from addressing these challenges, as doing so will help identify areas that require targeted safety interventions. Additionally, these datasets could be significantly important for the decision-making processes of intelligent automated vehicles, which, while generally safer, will likely still face risks in mixed environments with human-driven vehicles, pedestrians, and bicyclists.

Notably, this study uses historical crash records, constrained by limited data access, to validate the practicality of the theoretical framework for link-based crash cost estimates. While we do not anticipate that our key findings would change significantly with the latest updated dataset, given the recent statistics from crash reports and the consistency of previous studies over the past decade, we highly encourage future research to keep the data updated, preferably in a dynamic manner. This will better inform safety-related policy adjustments and enhance policy implementation.

Additionally, the estimates of crash frequency and severity, which serve as the foundation for the link-based crash cost analysis, are derived using negative binomial regression and ordered probit models. Currently, these models lack several important link attributes, such as the number of lanes, speed limits, curvature, and slope-factors that are anticipated to enhance predictive accuracy. While this limitation is not significant for the case of the Twin Cities, which are predominantly flat with well-maintained roads and effective lighting, it would be beneficial for future studies, especially those applying our framework in areas with varying slopes and road conditions, to collect this data for more reliable estimates.

Finally, for the unit cost specification, the framework employs Parry^14^’s settings for the external proportion of crash costs, which are derived from plausible yet heuristic methods. Refinements to these settings would improve accuracy, but would require more detailed data, including information on driver behavior, insurance policies, crash records, responsibility distribution, vehicle types, and other relevant factors.

## Data Availability

The datasets used and/or analysed during the current study available from the corresponding author on reasonable request.

## Footnotes

1 All estimates from previous studies are converted to 2014 U.S. dollars
